# Current Challenges in the Management of Prolactinomas

**DOI:** 10.17925/EE.2015.11.01.39

**Published:** 2015-04-11

**Authors:** Dominique Maiter

**Affiliations:** Head, Division of Endocrinology; Professor of Medicine, Department of Endocrinology and Nutrition Cliniques Universitaires Saint-Luc, Université catholique de Louvain, Brussels, Belgium

**Keywords:** Pituitary tumour, prolactin, diagnosis, treatment

## Abstract

Although the diagnosis of prolactinoma is often straightforward and the treatment strategy has been well defined in recent guidelines, several challenging issues persist in their management. The differential diagnosis of a large pituitary tumour with moderately elevated prolactin (PRL) concentrations is sometimes difficult, and prolonged treatment with a dopamine agonist may be inappropriate when the diagnosis of a prolactinoma is not sufficiently well substantiated. Also, timely withdrawal of dopamine agonist treatment and the remaining indications of transsphenoidal surgery are still matters of debate. Last but not least, the management of resistant or aggressive prolactinomas remains a challenge for the clinician, especially when they occur in young patients.

Prolactinomas are the commonest pituitary tumours with a prevalence varying from 0.3 to 0.5 per 1,000 in the general population. This affection has a large female predominance and a median age of 30 years at diagnosis.^1^ Prolactinomas in men are characterised by a larger size and a higher frequency of compressive symptoms, invasiveness and resistance to therapy.^2^ Although the diagnosis of a prolactinoma is often straightforward and the treatment strategy has been well defined in recent guidelines,^3^ several challenging issues persist in the management of this pituitary tumour.

The *diagnosis* of prolactinoma is usually based on the concomitant observations of a persistent hyperprolactinaemia and a pituitary adenoma at magnetic resonance imaging (MRI) with a good correlation between hormone levels and tumour size.^1^ It is rarely confirmed by immunocytochemistry of the tumour, as most prolactinomas are never operated. Macroprolactinomas can usually be recognised with a high degree of confidence when prolactin (PRL) concentrations are above 200 μg/l (4,200 mU/l)^4,5^. In patients with histologically confirmed non-functioning pituitary macroadenoma and stalk compression, PRL concentrations usually remain lower than 150 μg/l.^6^ In cases of doubt (divergence between tumour size and PRL concentrations, cystic or necrotic pituitary macroadenoma with intermediate levels of PRL between 100 and 200 μg/l), a 3- to 6-month trial with a dopamine agonist (DA) may be helpful, as most prolactinomas will show some degree of shrinkage under medical therapy. A thyrotropin-releasing hormone (TRH) stimulation test usually is not helpful in such circumstances.

It may be even more difficult to formally confirm the diagnosis of a microprolactinoma. Symptoms of hyperprolactinaemia are not specific of a tumoral origin, MRI findings may be ambiguous, non-functional pituitary incidentalomas are frequent and several other causes of moderate hyperprolactinaemia must be ruled out. Among them, drug-induced hyperprolactinaemia is a common condition (see 7 for review), and assessment for the presence of macroprolactin is also highly recommended in patients with asymptomatic hyperprolactinaemia.^3^ Noticeably, most microprolactinomas will be symptomatic, readily visible on dedicated MRI as a lateral nodule with a different T1 or T2 intensity than normal pituitary tissue, and exhibit PRL concentrations >40 μg/l (twofold the upper limit of normal). The absence of one or more of these criteria is still compatible with the diagnosis, but the clinician must be aware of other aetiologies and consider surveillance or early withdrawal of DA treatment before embarking the patient on long-term therapy. Discordance between excellent hormonal control under DA therapy and ongoing tumoral expansion must also lead to re-evaluation of the diagnosis.

Medical *treatment* of prolactinomas with a DA is the cornerstone of therapy, and cabergoline (CAB) is the first choice due to its high efficacy and good tolerability profile.^3,4^ Even in the case of very large adenomas with suprasellar extension, CAB induces rapid tumour shrinkage and visual field improvement in 80–90% of patients.^5^ One remaining factor is to decide when treatment can be safely withdrawn. Discontinuation of DA therapy is indeed possible with reasonably good chances of remission in well-defined conditions:^8^ (a) the patient has been continuously treated for at least 2 to 3 years (probably more for macroprolactinomas); (b) a low PRL concentration has been obtained with a low dose of DA (<0.5 mg/week of CAB); (c) a disappearance or a more than 50 % reduction in the maximal tumoral diameter has been observed; and (d) there is no cavernous sinus invasion. When such criteria are met, prolonged remission is observed in about one-third of the patients, more often after the use of CAB in the context of microprolactinomas (50 %) than with bromocriptine or in macroprolactinomas.^8^ These patients require, however, longterm surveillance, and a loss of follow-up with later resurgence of the prolactinoma is a potential risk of such strategy. It is also possible to discontinue dopaminergic therapy after menopause^3^ or after one or more pregnancies, which seem to increase the likelihood of remission.^9^

Neurosurgical treatment of prolactinomas is less effective than medical therapy, and recurrence of hyperprolactinaemia is frequent (10–50 % of patients). Nevertheless, transsphenoidal surgery should be considered in symptomatic patients who cannot tolerate any of the currently available DAs or who are not responsive to maximally tolerated doses of DAs.^3^ Besides these classic indications and some acute complications, such as apoplexy or cerebrospinal fluid (CSF) leakage, new indications have emerged, such as young patients with a high likelihood of complete tumour resection who do not wish to take prolonged medical treatment^10^ or patients who require high doses of CAB, in whom surgical debulking may significantly improve post-operative hormonal control.^11^ It is essential that the surgical procedure be performed by an experienced pituitary neurosurgeon. In such centres, immediate post-operative remission rates of 70–90 % have been reported for microprolactinomas and of 30-50 % for macroprolactinomas (reviewed in 12).

Resistance to DA treatment is another therapeutic challenge, being defined by a failure to achieve normal PRL levels on maximally tolerated doses of DAs and/or failure to achieve a 50 % reduction in tumour size.^2–4^ This resistance is rare in microadenomas, more frequent in cases of macroprolactinomas (3–5 %) and quite characteristic of invasive tumours.^2^ Although a decreased expression of D2 receptor has been evidenced in resistant prolactinomas, the mechanisms are still not completely understood.^13^

Several therapeutic options may be considered in cases of DA resistance.^2,13^ If the patient is treated with another DA, it is recommended to switch to CAB. About 70–80% of patients resistant to bromocriptine may indeed achieve PRL normalisation on CAB.^3^ Next, standard doses of CAB may be increased stepwise to maximal tolerable doses. In a Japanese study,^14^ increasing the CAB dose up to 12 mg/week allowed the overcoming of DA resistance in a few patients, but perhaps at the price of long-term undesirable side effects. Therefore, in the absence of any intolerable symptoms or emergency situation, we rather recommend keeping the maximal dose at 3.5 mg/week, which may, in turn, lead to hormonal control in half of the cases after 15 to 72 months.^2^ Finally, the resistant prolactinoma is also a valuable indication for transsphenoidal neurosurgery, aiming at large tumoral debulking and improved postoperative medical control.^11^ Irradiation must be considered only in the rare patients who fail surgical treatment and harbour aggressive prolactinomas.^3^ It may require up to 20 years for maximal effects to be achieved, and normalisation of hyperprolactinaemia occurs in only one-third of cases^4^. Finally, in aggressive prolactinomas or PRL-secreting carcinomas, a last therapeutic option may be the use of temozolomide, an orally administered alkylating agent, which might reduce PRL hypersecretion and control tumour growth in about 50 % of cases.^15,16^

Although the risk of restrictive cardiac valve disease seems to be very low at conventional doses (≤1.0 mg/week) of CAB,^17^ long-term echocardiographic surveillance (baseline evaluation and biannual monitoring) is indicated in patients who chronically need higher doses of DAs. It is also important to keep in mind that true resistance of prolactinomas to adequate medical treatment is often associated with a more aggressive behaviour of the tumour. These patients must therefore be followed more closely in a specialised centre.

## References

[1] Schlechte J. A. (2003). Clinical practice. Prolactinoma. N Engl J Med.

[2] Delgrange E., Daems T., Verhelst J. (2009). Characterization of resistance to the prolactin-lowering effects of cabergoline in macroprolactinomas: a study in 122 patients. Eur J Endocrinol.

[3] Melmed S., Casanueva F. F., Hoffman A. R. (2011). Diagnosis and treatment of hyperprolactinemia: an Endocrine Society clinical practice guideline. J Clin Endocrinol Metab.

[4] Gillam M. P., Molitch M. E., Lombardi G. (2006). Advances in the treatment of prolactinomas. Endocr Rev.

[5] Maiter D., Delgrange E. (2014). The challenges in managing giant prolactinomas. Eur J Endocrinol.

[6] Karavitaki N., Thanabalasingham G., Shore H. C. A. (2006). Do the limits of serum prolactin in disconnection hyperprolactinaemia need re-definition? A study of 226 patients with histologically verified non-functioning pituitary macroadenoma. Clin Endocrinol.

[7] Molitch M. E. (2005). Medication-induced hyperprolactinemia. Mayo Clin Proc.

[8] Dekkers O. M., Lagro J., Burman P. (2010). Recurrence of hyperprolactinemia after withdrawal of dopamine agonists systematic review and meta-analysis. J Clin Endocrinol Metab.

[9] Domingue M. E., Devuyst F., Alexopoulou O. (2014). Outcome of prolactinoma after pregnancy and lactation: a study on 73 patients. Clin Endocrinol (Oxf).

[10] Kreutzer J., Buslei R., Wallaschofski H. (2008). Operative treatment of prolactinomas: indications and results in current consecutive series of 212 patients. Eur J Endocrinol.

[11] Primeau V., Raftopoulos C., Maiter D. (2012). Outcomes of transsphenoidal surgery in prolactinomas: improvement of hormonal control in dopamine agonist-resistant patients. Eur J Endocrinol.

[12] Maiter D., Primeau V. (2012). 2012 update in the treatment of prolactinomas. Ann Endocrinol (Paris).

[13] Molitch M. (2008). The cabergoline-resistant prolactinoma patient: new challenges. J Clin Endocrinol Metab.

[14] Ono M., Miki N., Kawamata T. (2008). Prospective study of highdose cabergoline treatment of prolactinomas in 150 patients. J Clin Endocrinol Metab.

[15] Raverot G., Sturm N., de Fraipont F. (2010). Temozolomide treatment in aggressive pituitary tumors and pituitary carcinomas: a French multicenter experience. J Clin Endocrinol Metab.

[16] Bengtsson D., Daa Schrøder H., Andersen M. (2015). Long-term outcome and MGMT as a predictive marker in 24 patients with atypical pituitary adenomas and pituitary carcinomas given treatment with temozolomide. J Clin Endocrinol Metab.

[17] Valassi E., Klibanski A., Biller B. M. (2010). Clinical Review: Potential cardiac valve effects of dopamine agonists in hyperprolactinemia. J Clin Endocrinol Metab.

